# Myotube/Adipocyte Powder-Enriched Alginate–Zein Hydrogels Support Myotube Alignment for 3D Myoblast Culture

**DOI:** 10.3390/foods15030522

**Published:** 2026-02-02

**Authors:** Jihad Kamel, Jun-Yeong Lee, Chandra-Jit Yadav, Sadia Afrin, Usha Yadav, Sung Soo Han, Kyung-Mee Park

**Affiliations:** 1 College of Veterinary Medicine, Chungbuk National University, Cheongju 28644, Republic of Korea; jihadshabaan@gmail.com (J.K.); dkujunyeong@naver.com (J.-Y.L.); chandrajityadav84@gmail.com (C.-J.Y.); sadiaafrin1406@gmail.com (S.A.); ushacj.23@gmail.com (U.Y.); 2 School of Chemical Engineering, Yeungnam University, Gyeongsan 38541, Republic of Korea; sshan@yu.ac.kr

**Keywords:** alginate, adipocytes, C2C12, cell powder, cell culture, cultured meat, cell alignment, zein

## Abstract

Recent advances in cultured-meat research emphasize the development of edible scaffolds that promote myogenic differentiation. Nonetheless, many materials provide only structural support and do not replicate native muscle or serve as alternatives to muscle–adipocyte co-culture, highlighting the need for cytocompatible, tissue-specific scaffolds. This study aimed to develop a composite alginate–zein (Algi/zein) hydrogel enriched with myotube (MP) and adipocyte (AP) powders to provide a structural, biochemical, and potentially cultured-meat hydrogel. Algi/zein hydrogels enriched with myotube (MP) and adipocyte (AP) powders were fabricated and evaluated for structural, cellular, and biochemical properties using C2C12 myoblasts cultured in 2D and 3D environments. Metabolite profiling was performed to evaluate the biochemical features. MP/AP incorporation generated extra cellular matrix (ECM)-like microstructures and significantly enhanced myotube alignment in Algi/zein scaffolds compared with MP/AP-free controls, increasing the proportion of axially aligned fibers by up to ~6-fold at a 1:1 AP:MP ratio. Organized myosin expression was observed, while metabolomic profiling indicated partial biochemical similarity to beef. Incorporating MP and AP into Algi/zein hydrogels enhanced myotube alignment and showed partial structural and biochemical similarity to native muscle tissue.

## 1. Introduction

The use of biomaterials to construct scaffolds is fundamental to cultivated meat engineering, as they provide the framework for cell adhesion, proliferation, differentiation, and tissue formation [1,2]. Among various options, protein-based scaffolds are particularly attractive due to their biocompatibility, bioactivity, and ability to mimic the native ECM [3]. Proteins such as gelatin, collagen, zein, soy protein isolate, and myofibrillar proteins offer bio-recognition sites that promote myoblast attachment and myogenic differentiation [4,5]. Soy protein and zein, for example, have been used to create edible, biodegradable scaffolds that support muscle cell alignment and maturation [6,7,8]. These protein-based scaffolds are food-grade, plant-derived, and tunable in biochemical and mechanical properties, making them ideal for producing structured cultured meat [1].

Biomaterial-based scaffolds have garnered increasing attention in cultured-meat and tissue-engineering applications due to their intrinsic biocompatibility, biodegradability, and ability to mimic the native ECM microenvironment [9]. Synthetic polymeric scaffolds often require surface modification to support cell attachment and may generate non-degradable residues. Naturally derived biomaterials provide inherent cell-adhesive motifs, facilitate nutrient diffusion, and degrade into biologically tolerated by-products [10]. In the context of cultured-meat production, food-grade biomaterials further offer advantages in regulatory acceptance, edibility, and consumer safety [3,11]. Moreover, biomaterial scaffolds can recapitulate the biochemical and mechanical cues of muscle ECM, thereby supporting myoblast alignment, fusion, and maturation more effectively than inert synthetic matrices [12,13]. These features make biomaterial-based scaffolds particularly suitable for engineering structured, edible muscle tissues for cultivated-meat applications.

Zein and Algi have emerged as complementary biomaterials for developing edible scaffolds in cultivated meat due to their biocompatibility, sustainability, and ability to support cell growth and tissue structuring. Zein, a prolamin protein extracted from maize, is hydrophobic with good film-forming capacity and mechanical strength, making it suitable for mimicking the fibrous texture of muscle tissue [14,15,16]. Its structural properties promote cell alignment and anisotropic muscle-like formation; however, its poor water solubility and limited bioactivity can hinder cell adhesion and proliferation [17]. To overcome these shortcomings, zein is often combined with hydrophilic components such as Algi to enhance cell–material interactions [18].

Algi, a naturally occurring anionic polysaccharide from brown algae, forms hydrogels under gentle conditions that maintain a hydrated, cell-supportive environment [19]. It exhibits excellent biocompatibility and tunable gelation through crosslinking with divalent cations such as Ca^2+^, and also serves as a versatile bioink for 3D bioprinting of muscle constructs [20]. However, native Algi lacks intrinsic adhesion motifs, which limits cell attachment and differentiation; this limitation can be addressed by combining it with bioactive proteins like zein or gelatin [21]. The synergistic integration of zein and Algi enhances myoblast adhesion, proliferation, alignment, and myotube formation, improving mechanical and biochemical performance compared to single-material scaffolds [18,22]. This hybrid approach provides a structurally robust and edible scaffold suitable for scalable cultured-meat production [6].

Previous studies have shown that incorporating zein into polysaccharide- or protein-based scaffolds markedly improves their suitability for muscle tissue engineering. For example, zein–chitosan and zein–gelatin composites exhibited enhanced mechanical strength and significantly improved C2C12 cell adhesion and alignment compared with chitosan- or gelatin-only [23,24,25]. Similarly, incorporation of Algi into collagen- or gelatin-based hydrogels improved water retention, nutrient diffusion, and cytocompatibility, resulting in higher cell viability and differentiation compared to single collagen or gelatin-matrices [26,27]. Cultured-meat development requires both muscle and fat cells as well as native-like tissue architecture, including aligned muscle fibers and adipose integration. Adipocytes are essential for reproducing the sensory attributes of traditional meat, including flavor, juiciness, and lipid composition [28,29]. Although co-culturing muscle and fat cells offers a biologically relevant strategy to replicate tissue complexity, their distinct metabolic and differentiation requirements pose challenges in vitro [1,3]. Achieving native-like myotube alignment also remains limited, as in vitro muscle cells often form disorganized aggregates in the absence of appropriate guidance cues [30,31]. While methods such as micropatterning, aligned bioprinting, and dynamic culture have been developed, these strategies are difficult to scale and standardize [32,33,34,35]. To address these challenges, recent studies have explored modular tissue engineering approaches, including the separate supplementation of adipose and muscle components [36]. However, many edible scaffold systems still primarily provide physical support and lack integrated lineage-specific bioactivity required for effective muscle tissue mimicry [28,37]. Together, these limitations underscore the need for strategies such as the incorporation of cell-derived components that enable scalable, bioactive, and food-grade scaffold systems for fully biomimetic cultured meat [36].

Despite recent advances in edible scaffold development, several critical limitations remain. First, most food-grade scaffolds primarily provide mechanical support and lack lineage-specific biochemical bioactivity capable of simultaneously delivering myogenic and adipogenic cues within a single matrix [38]. Second, the induction of myotube alignment typically relies on external patterning strategies, molds, or mechanical stimulation, which limits fabrication simplicity and scalability [39]. Third, many alignment-directing scaffolds lack comprehensive food-grade biochemical validation, restricting their translational relevance to cultured-meat applications [38,39]. Collectively, these limitations underscore the need for edible scaffold systems that integrate intrinsic bioactivity, scalable self-alignment capacity, and food-grade biochemical functionality.

Accordingly, the primary objective of this study was to evaluate whether the incorporation of MP/AP into Algi/zein hydrogels enhances myoblast alignment compared with MP/AP-free scaffolds. As a secondary objective, we further assessed whether MP/AP-derived cues confer muscle-like biochemical characteristics.

We hypothesized that incorporation of MP and AP powders into Algi/zein hydrogels would provide intrinsic biochemical and topographical cues sufficient to enhance myotube alignment without the need for external patterning, while also introducing muscle-like biochemical features.

## 2. Materials and Methods

### 2.1. Cell Culture

Mouse C2C12 cells (CRL-1772, ATCC, Manassas, VA, USA) were used in co-culture in both 2D and 3D configurations. The growth media consisting of high-glucose DMEM and 2mM L-glutamine (Thermo Fisher, Waltham, MA, USA), supplemented with 10% (*v*/*v*) fetal bovine serum (FBS) (Cytiva, Marlborough, MA, USA) and 1% (*v*/*v*) antibiotic-antimycotic (ABAM) (WelGene, Gyeongsan, The Republic of Korea) at 37 °C with 5% CO_2_. For cell differentiation, a DMEM medium containing 2% horse serum (HS) and 1% antibiotic-antimycotic was used. The differentiation process was conducted for 10 days, with the media changing every two days.

Murine preadipocyte was isolated according to a previous report [40]. Briefly, preadipocytes were isolated from fat tissue (0.4 g) at the posterior inguinal region of 6–12-week-old mice. The excised tissue was disinfected with iodine solution (2%) for 5 min. The tissue was finely minced and digested in a solution of collagenase from Clostridium histolyticum (Sigma Aldrich, St. Louis, MO, USA) (1 mg/mL) at 37 °C for 30–40 min with constant agitation. The digested mixture was filtered through a 100 µm cell strainer to remove debris and centrifuged at 500× *g* for 5 min to pellet the stromal vascular fraction (SVF). The SVF pellet was resuspended in a T75 culture dish (SPL, Gyeonggi, Republic of Korea) with growth medium composed of DMEM F-12 (Thermo Fisher) supplemented with 20% FBS and 1% ABAM, then plated onto tissue culture-treated dishes. Cells were expanded until 70–80% confluency with medium changes every 2–3 days. For adipogenic differentiation, confluent cells were treated with induction medium containing 0.5 mM IBMX, 5 µM dexamethasone, 1 µg/mL insulin, and 1 µM rosiglitazone for 2 days (Sigma Aldrich), followed by maintenance medium (DMEM F-12 with 20% FBS and 1 µg/mL insulin) for an additional 10 days, with media changes every 2–3 days. Lipid accumulation confirmed successful adipogenic differentiation.

Oil Red O staining was carried out using Oil Red O solution (Sigma-Aldrich). Following the removal of maintenance medium, cells were fixed with 4% paraformaldehyde (PFA) for 30 min. The fixative was then discarded, and the cells were gently washed 2–3 times with DW. Subsequently, Oil Red O working solution (3:2 ratio with DW) was applied to the cells and incubated for 1 h. After staining, the solution was removed, and the cells were washed gently 2–3 times with DW. Finally, the cells were covered with DW to prevent drying and observed under a microscope.

Murine preadipocytes were used only as an in vitro research model to evaluate scaffold bioactivity and adipogenic differentiation, and this study is not intended for direct food production or regulatory submission.

### 2.2. Cell Powder Preparation

Cell Powder Meat (CPM) was developed previously as a high-protein, meat-flavored food produced without 3D scaffolds and with reduced serum, compared to conventional methods [36]. Here, we optimized the cell powder technology with minor alterations. Briefly, each C2C12 and murine preadipocyte was cultured separately using 10 dishes of a 150 × 25 mm cell culture dish (SPL), and the differentiation process lasted for 10 days. The procedure of cell growth was described in Section 2.1. The differentiation media were removed, and the cells were washed with 1× phosphate-buffered saline (PBS). Then, the cells were gently detached using cell scrapers, starting from the edge and working inwards. The mass of detached cells was collected using DW and transferred to a 15 mL conical tube. Centrifugation was conducted for 4 min at 1200× *g* force to form the cell pellet. The supernatant was removed, and the pellets were frozen at −20 °C for one day and then freeze-dried at −50 °C for another day. The collected mass of cell powder was sterilized under UV light for 3 h before dissolving in media. Finally, MP and AP were prepared from differentiated C2C12 myotubes and adipocytes, respectively, and used as powdered supplements for subsequent scaffold composition and cell culture experiments.

Cell powders were prepared in two independent batches using the same protocol. In each batch, MP and AP were produced separately from 10 dishes (150 × 25 mm) per cell type. The final dry powder yield was consistent between batches (~1.25–1.30 g per batch).

### 2.3. Biocomposite Algi/Zein Hydrogel

#### 2.3.1. Algi/Zein Hydrogel Preparation

The alginate and zein concentrations were selected based on the reported formulation [41]. This composition ensured stable gelation and homogeneous mixing for cell culture. Briefly, 0.4 g of zein (Sigma Aldrich) was dissolved in 20 mL of 75% ethanol, then 10 mL of the resolved zein was added to 30 mL of 1% Algi (Sigma Aldrich) dissolved in 50 °C distilled water (DW). Ethanol was used only as a transient solvent and was removed before analysis. The homogenate of Algi/zein hydrogel was stirred using a magnetic bar at 50 °C. Then, 40 mL of the Algi solution was added to the homogenate, reaching a concentration of 0.28%. The Algi/zein hydrogel was preserved in the refrigerator for 7 days.

#### 2.3.2. Cell Seeding

After preparing the Algi/zein hydrogel, C2C12 cells were seeded on top with 5 × 10^4^ cells per 1 mL of hydrogel, making a 1 mm thickness. The cross-linking process was determined using 2% calcium chloride (CaCl_2_) (Sigma Aldrich) in DW for 5 min, and then the scaffold was washed gently with 1× PBS. The growth media DMEM containing 10% FBS and 1% ABAM (3 mL) was used to cover the scaffold in a 35 × 10 mm dish. Both types of MP and AP were established to study their effect on the 2D culture of C2C12 cells. First, we determined the effect of different concentrations of MP on C2C12 cytotoxicity, proliferation, and differentiation. The different concentrations were added in the proliferation and differentiation media with 0.01, 0.05, 0.1, 0.2, 0.3, 0.4, 0.5, 1.0, 1.3, and 1.5 μg/μL. The control group was determined without adding MP to the growth media. After successful optimization of MP concentration, we determined different ratios of AP:MP as 1:0, 1:1, 2:1, 4:1, and 6:1, respectively. For probe powder dissolving, both were kept at −20 °C for 1 h (h), and then MP was ground with ~1 mL of media before determining the different concentrations. While AP was dissolved in 0.1% Dimethyl sulfoxide (DMSO) (Sigma Aldrich) before preparing AP:MP ratios. The final DMSO concentration in all culture conditions did not exceed 0.1% (*v*/*v*).

In the 3D experiments, AP and MP powders were mixed at a 1:1 ratio and incorporated into the Algi/zein hydrogel (Algi/zein ^(AP:MP)^). A 1 mL aliquot of the hydrogel was transferred into a 4-well culture plate and combined with 5 × 10^4^ C2C12 cells. The constructs were subsequently crosslinked using 2% CaCl_2_ in distilled water for 5 min. After cross-linking, excess CaCl_2_ was removed, and the scaffold was washed once with 1× PBS, and then the media was added (DMEM containing 10% FBS and 1% ABAM). C2C12 cells were proliferated through Algi/zein ^(AP:MP)^ for 3 days and differentiated for 10 days, with media changing every two days.

#### 2.3.3. Physical Characteristics

Briefly, the swelling ratio (*SR*) of hydrogels with a uniform thickness of 7 mm was evaluated at 37 °C and 5% CO_2_. Before incubation, each scaffold was freeze-dried at −50 °C for 24 h, and its initial dry mass (*W*0) was recorded as the baseline reference weight. Hydrogels were then incubated in fresh culture medium for 0 (immediately post-gelation), 1, 3, 5, 7, and 10 days, with medium replaced every two days. At each point, independent samples were gently blotted to remove excess surface liquid, and their wet mass (*Wt*) was recorded. The swelling ratio was calculated using the standard formula [42]:

𝑆𝑅(%) = *𝑊*𝑡 − *𝑊*0/*𝑊*0 × 100.

To avoid circularity between swelling and degradation measurements, separate sets of samples were used for degradation analysis. For degradation testing, the baseline dry mass (*𝑊*0) is determined as described above. At each time point, independent samples were removed from the medium, dried at 60 °C for 4 h, and the remaining dry mass (*𝑊*) was recorded [43].

The degradation rate (%) = *𝑊*0 − *𝑊*/*𝑊*0 × 100.

The experimental unit was one individual hydrogel scaffold. Swelling and degradation were measured using *n* = 8 independent scaffolds per group per time point (destructive sampling).

#### 2.3.4. Scanning Electron Microscopy (SEM)

Pore disruption on both the outer and inner surfaces of the scaffolds was assessed using SEM analysis. The samples were pretreated and dried at room temperature (RT) for 12 h, followed by gold coating via sputter deposition. Surface morphology was analyzed using SEM (Gemini 560, Oberkochen, Germany). Images captured at 10,000× magnification were used to quantify the pore area percentage and average pore size using ImageJ software (version 1.47). For each scaffold, pore quantification was performed using three SEM images from three different, non-overlapping fields of view (*n* = 3 fields per scaffold). Representative images were selected from these fields based on typical morphology.

#### 2.3.5. Cytotoxicity

The cytotoxic effects of MP and AP at the concentrations described above were evaluated using the Cell Counting Kit-8 (CCK-8; Sigma-Aldrich) on days 1, 3, and 5 of culture. At each time point, a 10% (*v*/*v*) CCK-8 solution was added to the culture medium and incubated for 4 h. Following incubation, 100 µL of supernatant from each sample was transferred to a 96-well plate, and absorbance was measured at 450 nm using a NanoQuant plate reader (TECAN Ltd., Männedorf, Switzerland).

Cell viability was further assessed using the Live/Dead™ Cell Imaging Kit (Thermo Fisher Scientific). Briefly, 40 µL of the Live/Dead staining solution was added to the conditioned medium and incubated for 30 min, after which cells were imaged using fluorescence microscopy. Cells cultured without MP or AP supplementation (0% powder) served as the positive control, while fresh culture medium was used as the negative control. Absorbance values were not normalized to surface area because all groups were tested under identical culture conditions using the same seeding density and culture volume.

#### 2.3.6. Immunofluorescence Staining

To assess C2C12 proliferation and differentiation, the expression levels of paired box 7 (PAX-7), Myo-d, desmin, and myosin antibodies were evaluated. The hydrogel was washed with PBS and fixed with BIOFIX HD (BIOGNOST, Zagreb, Croatia) for 20 min. Following fixation, the hydrogels were treated with 0.2% Triton X-100 (SAMCHUN, Ulsan, South Korea) for 10 min and subsequently washed with phosphate-buffered saline with Tween-20 (PBST). A solution of 1% bovine serum albumin (BSA, Sigma Aldrich) dissolved in 1X PBST was applied for 30 min. After removing the BSA, each hydrogel was stained with PAX-7 (1:100, Invitrogen, Waltham, MA, USA), Myo-d (1:100, Thermo Fisher Scientific), α-desmin (1:100, Sigma Aldrich), myoglobin antibody (1:100, Thermo Fisher Scientific), and anti-myosin skeletal muscle antibodies (1:100, Sigma Aldrich) for 1 h at RT. The samples were then washed three times with PBST and incubated with the secondary antibody, goat anti-mouse IgG (1:500, Invitrogen), for 1 h. Finally, the samples were washed twice with PBST, and the cell nuclei were stained with DAPI (1:1000) (Sigma Aldrich) for 5 min. Desmin images were utilized to quantify the relative frequency (%) of myotube orientation using the Directionality plugin in ImageJ (version 1.47).

#### 2.3.7. Fourier Transform Infrared Spectrometer (FTIR)

FTIR spectroscopy of Algi/zein and Algi/zein ^(AP:MP)^ hydrogel scaffolds was performed using a Cary 670 bench spectrometer coupled with a Cary 620 microscope (Agilent Technologies, Santa Clara, CA, USA). Samples were measured in ATR mode over the range 500–4500 cm^−1^ with a spectral resolution of 4 cm^−1^, and 32 scans per sample were recorded (number of scans averaged for each spectrum: 32). Hydrogels were measured directly without additional pre-treatment. Spectra were plotted as transmittance versus wavenumber using GraphPad Prism software. No baseline correction or Amide I deconvolution was applied. Observed peaks were interpreted based on standard band assignments for proteins, lipids, and carbohydrates [44].

#### 2.3.8. Flavor Analysis

Metabolite extraction and analysis were conducted to assess the biochemical composition of Algi/zein, Algi/zein ^(AP:MP)^ scaffolds, and commercial beef samples. We determined the flavor analysis that was previously developed for CPM proteins with minor alterations [36]. Briefly, after 10 days of cell differentiation through the scaffolds, samples (lyophilized for 24 h) underwent solvent-based metabolite extraction following standard protocols for polar and semi-polar compounds. The extracts were derivatized with methoxamine hydrochloride in pyridine, followed by treatment with N, O-bis(trimethylsilyl)trifluoroacetamide (BSTFA) to improve metabolite volatility and thermal stability. Metabolite profiling was carried out using a gas chromatography-mass spectrometry (GC-MSD) system (Agilent 8890/5977B GC/MSD, Agilent Technologies, CA, USA) equipped with an HP-5MS capillary column (30 m × 0.25 mm, 0.25 µm film thickness). A 1 µL injection volume was used in splitless mode, with helium as the carrier gas at a constant flow rate of 1.0 mL/min. The oven temperature program was as follows: initial temperature at 60 °C (held for 1 min), ramped to 325 °C at 10 °C/min, and held for 10 min. Injector and transfer line temperatures were maintained at 250 °C and 280 °C, respectively. The mass spectrometer operated in electron impact (EI) ionization mode at 70 eV, scanning a mass range of *m*/*z* 50–600. Identifications were reported at MSI Level 3. Hierarchical clustering heatmaps were used for data visualization.

#### 2.3.9. Statistical Analysis

All quantitative data are presented as mean ± standard error (SE). Statistical analyses were performed using GraphPad Prism version 8.0.1.244 (64-bit, Windows). Data were analyzed using two-way ANOVA with repeated measures. When missing data points were present, mixed-effects models (REML) were applied. Post hoc comparisons were corrected using Holm–Šidák’s multiple-comparison tests, as appropriate. For each condition, *n* represents the number of individual hydrogel samples (scaffold replicates) analyzed per group (*n* = 4–8), as indicated in the figure legends. Adjusted *p*-values (q-values) < 0.05 were considered statistically significant.

## 3. Results

### 3.1. Physical Properties of Algi/Zein Hydrogel Scaffold

**Figure 1:**
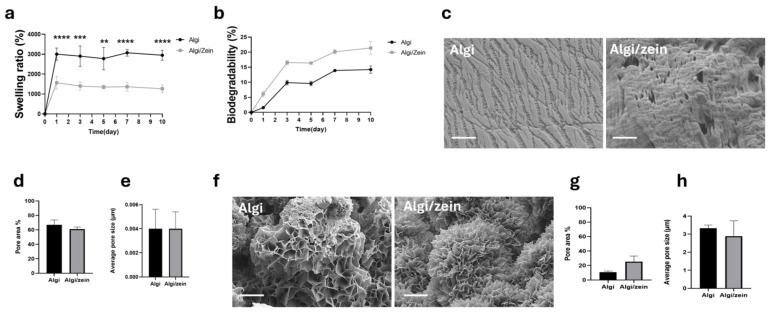
Evaluation of the physical properties and microstructure of alginate (Algi) and alginate/zein (Algi/zein) composite hydrogels. (**a**) Swelling behavior (%) of Algi and Algi/zein hydrogels over 10 days. Algi/zein hydrogels exhibited a lower swelling ratio compared to pure Algi hydrogels. (**b**) Biodegradability (%) over 10 days, showing degradation in Algi/zein hydrogels. (**c**) SEM outer surface morphology of Algi (**left**) and Algi/zein (**right**) hydrogels, revealing that zein incorporation modified surface texture without significantly altering pore patterns. Scale bar 10 μm. (**d**) Quantitative comparison of surface average pore size between Algi and Algi/zein hydrogels (*n* = 3). (**e**) Outer surface pore area percentage shows no significant change after zein addition. (**f**) SEM images of internal morphology of Algi (**left**) and Algi/zein (**right**) hydrogels, indicating porous inner structure of both hydrogels due to water absorption. Scale bar 500 nm. (**g**) Quantification of internal average pore size showing comparable values between Algi and Algi/zein. (**h**) Internal pore area percentage of Algi and Algi/zein hydrogels, demonstrating retained porosity despite zein incorporation. All data are presented as mean ± SE. *n* = 4–8. Data was analyzed using a two-way ANOVA, followed by Šidák corrected post hoc comparisons between scaffolds at each time point. Significance was denoted by ** *p* < 0.01, *** *p* < 0.001, and **** *p* < 0.0001.

Over 10 days, Algi/zein hydrogel exhibited significantly lower swelling (1265.41 ± 191.67) compared to Algi hydrogel (2947.92 ± 252.95) (Figure 1a), indicating reduced water uptake following incorporation of zein. This reduced swelling is consistent with the hydrophobic nature of zein and suggests that zein incorporation modulates the hydration behavior of the Algi-based scaffold.

Biodegradation of Algi and Algi/zein hydrogels was evaluated over the same 10-day period. Although Algi/zein hydrogels showed a higher mean biodegradation percentage (21.38 ± 2.16%) compared with Algi (14.21 ± 1.23%), no statistically significant differences were observed at any time point (Figure 1b). These results indicate that incorporation of zein alters swelling behavior without substantially affecting the overall degradation profile of the Algi-based hydrogel.

SEM analysis revealed that both Algi and Algi/zein hydrogels exhibited a narrow and comparable pore distribution on the outer surface (Figure 1c). Quantitative image analysis showed that the Algi/zein displayed a pore area of 61.00 ± 2.93% and an average pore size of 0.004 ± 0.001 μm, which were not significantly different from those of the Algi hydrogel (66.87 ± 6.94% pore area and 0.004 ± 0.001 μm average pore size) (Figure 1d,e). That suggesting the incorporation of zein does not compromise the outer-surface porosity of the Algi-based scaffold.

Analysis of the internal morphology showed that both Algi and Algi/zein hydrogels exhibited a wider pore distribution on the inner surface compared with the outer surface, likely due to water adsorption (Figure 1f). Quantitative analysis showed that the Algi/zein exhibited an inner-surface pore area of 25.31 ± 7.90% and an average pore size of 2.89 ± 0.85 μm, compared with Algi hydrogel (10.78 ± 1.59% and 3.33 ± 0.18 μm) (Figure 1g,h). These findings suggest that incorporation of zein does not alter the internal porosity of the Algi-based scaffold.

### 3.2. Algi/Zein Hydrogel Scaffold for C2C12 Growth

**Figure 2:**
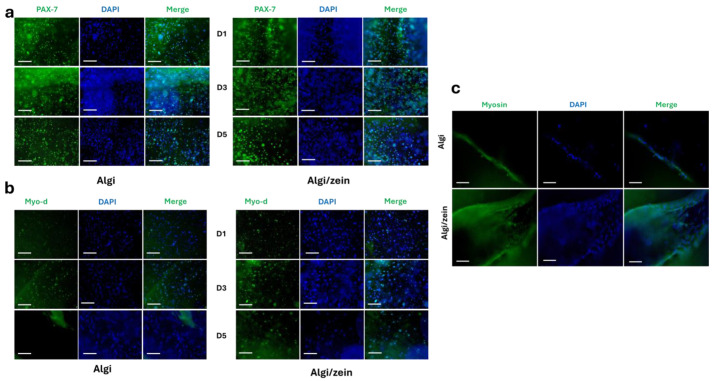
Evaluation of Proliferation and Differentiation of C2C12 Cells within Alginate-zein (Algi/zein) hydrogels. (**a**) Immunofluorescence staining for the proliferation marker PAX-7 showed high expression at day 1 and day 3, and markedly decreased by day 5, suggesting limited proliferation in the Algi/zein hydrogel compared to the control Algi hydrogel. (**b**) MyoD immunostaining revealed progressive expression from day 1 to day 5. Scale bar 200 μm. (**c**) Immunofluorescence for Myosin (a late differentiation marker) showed well-formed, multinucleated myotubes in the Algi/zein hydrogel, suggesting terminal differentiation. Scale bar 100 μm.

Immunostaining of the proliferation marker PAX-7 confirmed the presence of proliferative C2C12 cells in both Algi/zein and Algi hydrogels during the first 5 days (Figure 2a). During the proliferation phase, MyoD staining indicated initiation of myogenic differentiation on days 3 and 5 (Figure 2b). After differentiation induction, myosin staining showed multinucleated myotube formation in both hydrogel groups (Figure 2c). Overall, these results indicate that both Algi/zein and Algi hydrogels supported C2C12 proliferation and myogenic differentiation.

### 3.3. Cell Powder Formation

**Figure 3:**
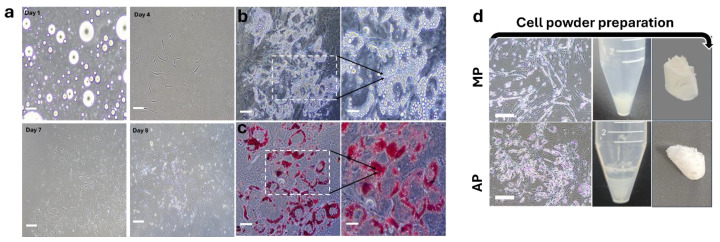
Isolation of murine preadipocytes and preparation of adipocyte (AP) and myotube powders (MP). (**a**) Brightfield images showing the morphological progression of isolated murine preadipocytes over 9 days. On day 1, cells appear as small round structures, transitioning into a fibroblast-like morphology by day 4, and reaching full confluency by day 9. Scale bar 200 μm. (**b**) Brightfield images during adipogenic differentiation, where lipid droplet accumulation is observed in mature adipocytes. Scale bar 100 μm, and 50 μm in magnified insets (**c**). Oil Red O staining confirms lipid accumulation within differentiated adipocytes, with red-stained lipid droplets indicating successful adipogenesis. (**d**) Representative images showing the preparation workflow and final product MP and AP. C2C12 myotubes and mature adipocytes were harvested, pelleted, frozen at −20 °C, lyophilized, and sterilized under UV light to obtain cell-derived powder. Scale bar 200 μm.

The adipogenic differentiation process was monitored from Day 1 to Day 9 under adipogenic induction. On Day 1, cells appeared as rounded structures in suspension. By Day 4, they adhered and gradually adopted an elongated fibroblast-like morphology, forming a dense monolayer by Day 9, indicating successful proliferation (Figure 3a). Lipid droplet accumulation was observed by phase-contrast microscopy, as dense intracellular vacuoles were visible within differentiated cells, signifying the onset of adipogenesis (Figure 3b). This was further confirmed by Oil Red O staining, which revealed red-stained lipid droplets, validating the functional maturation of adipocytes (Figure 3c). Differentiated C2C12 and murine adipocyte cells were harvested by centrifugation and lyophilization to form compact, tissue-like pellets (Figure 3d). These final constructs exhibited a dense powder-like appearance, demonstrating the feasibility of this method for producing high-protein and fat-rich cell powders as potential food components.

### 3.4. Impact of MP on C2C12 Cell Growth

**Figure 4:**
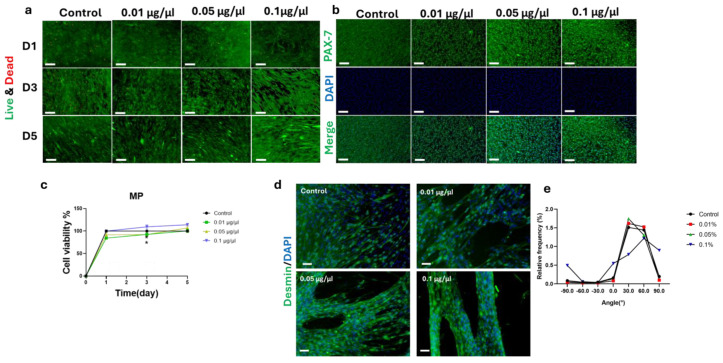
Effects of myotube cell powder (MP) on growth, viability, and differentiation of C2C12 cells. (**a**) Live/Dead staining of C2C12 cells cultured with MP at 0.01, 0.05, and 0.1 μg/μL shows high cell viability and no cytotoxicity. Scale bars 100 μm. (**b**) Immunostaining for PAX-7 (green) and DAPI (blue) indicates no negative impact of MP after 3 days of C2C12 cell proliferation. Scale bars 100 μm. (**c**) CCK-8 assay reveals that cell viability remains above 90% across all MP concentrations, with the highest viability observed at 0.1 μg/μL on day 5. The control group represents plain media. (**d**) Immunofluorescence staining for desmin (green) and DAPI (blue) shows that 0.1 μg/μL MP promotes myotube formation and alignment. Scale bars 100 μm. (**e**) Quantification of myotube alignment indicates the highest frequency at 0° orientation in the 0.1 μg/μL group, suggesting improved structural organization. All data are presented as mean ± SE. n = 4–8. Data was analyzed using a two-way ANOVA, followed by Šidák corrected post -hoc comparisons between concentrations at each time point. Significance was denoted by * *p* < 0.05.

The effects of MP on C2C12 cell viability and proliferation were evaluated in 2D culture using Live/Dead staining and CCK-8 assays. Live/Dead staining demonstrated high cell viability across all MP concentrations tested (0.01, 0.05, and 0.1 μg/μL), indicating cytocompatibility of the MP-enriched environment (Figure 4a). The immunofluorescent staining of PAX-7 indicates the maintenance of proliferated C2C12 cells after 3 days (Figure 4b). Consistently, CCK-8 analysis showed no cytotoxic effects at any concentration, with a non-significant increase in viability observed at 0.1 μg/μL MP (114.16 ± 3.27%) compared with 0.05 μg/μL (100.42 ± 3.99%) and 0.01 μg/μL (106.94 ± 4.97%) (Figure 4c). In contrast, higher MP concentrations (>0.1 μg/μL; 0.2, 0.3, and 0.4 μg/μL) induced marked cytotoxicity and significant cell death over 5 days (Figure S1a,b), defining 0.01–0.1 μg/μL as the working concentration range.

Myogenic differentiation was assessed by desmin immunostaining. MP supplementation up to 0.1 μg/μL increased myotube formation and improved myotube organization compared with the control group, which showed scarce and disorganized desmin-positive fibers (Figure 4d and Figure S2). The 0.05 and 0.1 μg/μL MP groups exhibited denser and more aligned myotubes, indicating enhanced structural maturation. Myotube orientation was further quantified (Figure 4e), showing that 0.1 μg/μL MP resulted in a greater proportion of parallel-aligned myotubes. Collectively, these findings indicate that MP at 0.1 μg/μL supports cytocompatibility and promotes organized desmin-positive myotube formation.

### 3.5. Impact of AP on C2C12 Cell Growth

**Figure 5:**
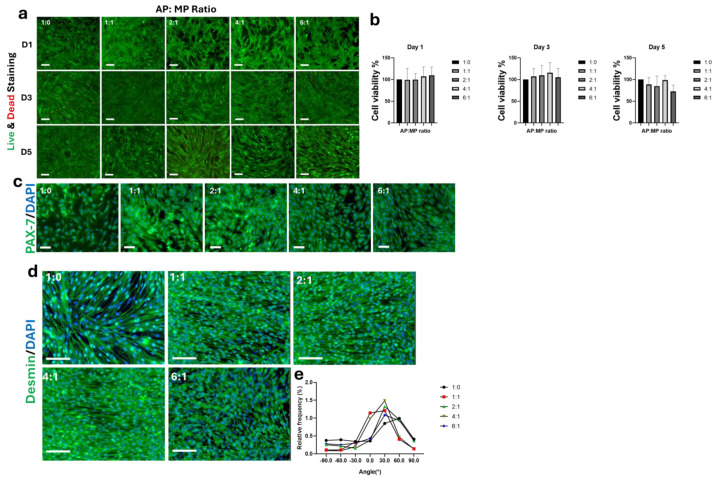
Effect of adipocyte-to-myotube powder (AP:MP) ratios on C2C12 cell growth, proliferation, and differentiation. (**a**) Live/Dead staining demonstrates minimal cytotoxicity across all tested AP:MP ratios (1:0, 1:1, 2:1, 4:1, 6:1), indicating that the powders are biocompatible. Scale bars 100 μm. (**b**) CCK-8 assay shows sustained C2C12 cell viability (80–100%) under all treatment conditions. Data are presented ± SE. (*n* = 8). (**c**) Immunostaining for PAX-7 (green) and DAPI (blue) confirms that none of the tested AP:MP ratios negatively impact C2C12 proliferation. Scale bars 100 μm. (**d**) Immunostaining for desmin (green) and DAPI (blue) indicates successful myogenic differentiation at all ratios, with increasing myotube alignment observed from 1:0 to 1:1 and beyond. Scale bars 100 μm. (**e**) Myotube alignment analysis shows the highest alignment frequency at 0° orientation in the 1:1 group, suggesting this ratio promotes optimal structural organization of C2C12-derived myotubes.

We studied the influence of AP:MP on cell viability, proliferation, alignment, and differentiation across various ratios. First, Live/Dead staining of C2C12 cells treated with 1:0, 1:1, 2:1, 4:1, and 6:1 AP:MP, respectively. AP:MP-treated conditions maintain high viability across all ratios (Figure 5a). Quantification of cell viability using CCK-8 showed a non-significant difference compared to control (1:0), suggesting a non-cytotoxic effect of AP:MP ratios on C2C12 growth (Figure 5b). The Immunostaining for PAX7 expression was preserved across all ratios, indicating maintenance of myogenic potential (Figure 5c).

Immunostaining for desmin was used to visualize cytoskeletal organization and myogenic differentiation. MP: AP -treated cells (1:1, 2:1, and 4:1) exhibited more uniform desmin organization compared with control (1:0), whereas a higher MP: AP ratio (6:1) was associated with disrupted desmin integrity (Figure 5d). Myotube alignment (desmin^+^ myotubes) was analyzed from −90° to 90°, and the fusion index at 0° was used as the primary orientation endpoint. All MP/AP-enriched media showed a general alignment shift toward ~30° angle. Among these, the 1:1 AP:MP formulation exhibited the greatest enrichment of axially aligned fibers (0° ± 10°). Specifically, the 1:0, 1:1, 2:1, 4:1, and 6:1 AP:MP groups showed 125%, 617%, 136%, 512%, and 169%, respectively (Figure 5e). Overall, under the tested conditions, the 1:1 AP:MP ratio showed the highest enrichment of axially aligned myotubes while maintaining cell viability, suggesting it as a suitable working ratio for subsequent experiments.

### 3.6. Establishment of Algi/Zein ^(AP:MP)^ Composite Scaffold

#### 3.6.1. Coating Algi/Zein Hydrogel with AP:MP Components

**Figure 6:**
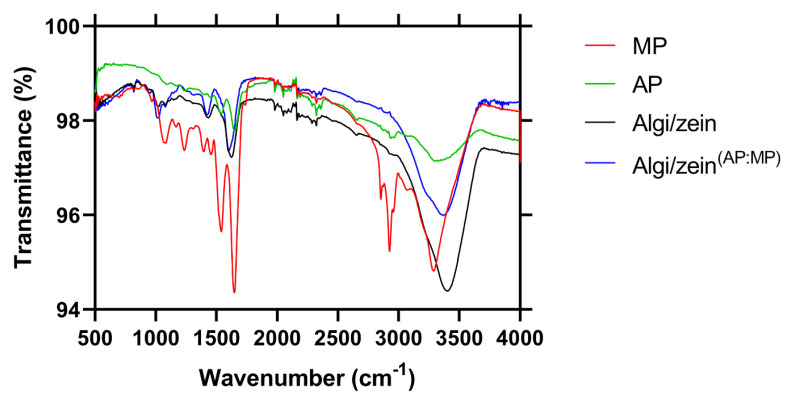
FTIR spectra on Myotube Powder (MP), Adipocyte Powder (AP), and Alginate–zein (Algi/zein) hydrogel supplemented with both AP and MP (Algi/zein AP:MP). The red spectrum corresponds to pure MP, the green spectrum represents pure AP, and the blue spectrum corresponds to the Algi/zein AP:MP. Distinct absorption bands are observed in the regions corresponding to proteins (amide I and II bands near 1650 cm^−1^ and 1540 cm^−1^), lipids (C–H stretching between 2800 and 3000 cm^−1^), and carbohydrates (C–O and C–O–C stretching in the fingerprint region below 1200 cm^−1^). The hydrogel spectrum shows characteristic features from both AP and MP, indicating successful incorporation of cellular components into the scaffold matrix. The control group represents plain Algi/zein hydrogel (Black spectrum).

FTIR spectroscopy was employed to characterize the chemical features and compositional differences in Algi/zein and Algi/zein ^(AP:MP)^ hydrogels (Figure 6). All spectra showed broad absorption bands around 3270–3300 cm^−1^, indicating O–H and N–H stretching vibrations associated with proteins and water. The MP spectrum displayed prominent peaks at ~1650 cm^−1^ (amide I, C=O stretching) and ~1540 cm^−1^ (amide II, N–H bending), consistent with protein presence [44]. In contrast, the AP spectrum showed weaker amide bands but more pronounced absorptions in the region of 1200–2000 cm^−1^, likely associated with C–O stretching of carbohydrates and lipid-related groups. The control sample (Algi/zein) exhibited lower overall intensity and fewer distinct absorption peaks. These spectral features reflect the unique molecular compositions of the Algi/zein ^(AP:MP)^ hydrogel. MP is protein-dominant, whereas AP shows lipid and carbohydrate signatures (Table 1).

#### 3.6.2. C2C12 Growth Through Algi/Zein ^(AP:MP)^ Scaffold

**Figure 7:**
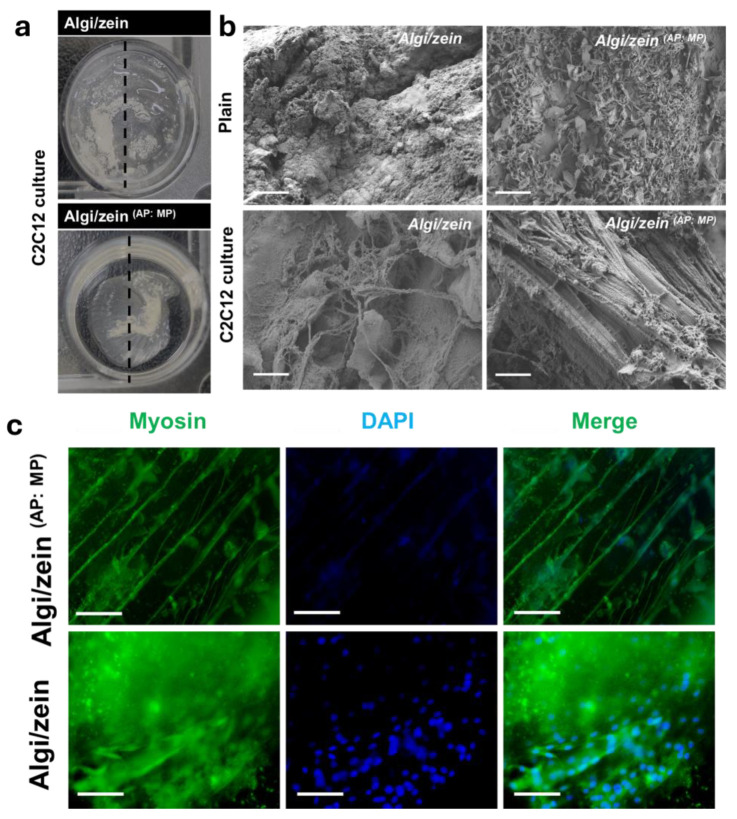
Morphological and immunofluorescence analysis of Alginate-zein (Algi/zein) scaffold supplemented with Adipocyte (AP) and Myotube Powder (MP) (Algi/zein AP:MP). (**a**) Macroscopic top-view images show visible differences in scaffold surface morphology between Algi/zein and Algi/zein ^(AP:MP)^ after 10 days of cell differentiation. Algi/zein hydrogel showed a swelled and smooth surface compared to the straightened and shrunken morphology of Algi/zein (^AP:MP)^ scaffold. (**b**) Scanning electron microscopy (SEM) images of Algi/zein and Algi/zein ^(AP:MP)^ scaffolds reveal distinct microstructural features. The plain Algi/zein scaffold exhibits a relatively compact and amorphous structure. In contrast, the Algi/zein ^(AP:MP)^ scaffold shows fibrous orientation and embedded powder residues, indicating successful integration of AP and MP components. The cultured Algi/zein scaffold exhibits an ECM secreted during cell differentiation. While the culture Algi/zein ^(AP:MP)^ showed a cigar, aligned myotube formation. Scale bars 10 μm. (**c**) Immunofluorescence staining for myosin (green) and nuclei (DAPI, blue) demonstrates enhanced myogenic differentiation on the Algi/zein ^(AP:MP)^ scaffold compared to the Algi/zein group. Algi/zein ^(AP:MP)^ showed myotubes in longitudinal alignment. Scale bars 100 μm.

After 10 days of C2C12 differentiation within the hydrogel scaffolds, morphological characterization showed differences between Algi/zein and Algi/zein ^(AP:MP)^ scaffolds. The Algi/zein ^(AP:MP)^ scaffold exhibited a smoother and more striated surface compared with the relatively diffuse Algi/zein hydrogel (Figure 7a), indicating increased structural compactness after AP/MP incorporation. SEM imaging supported these observations (Figure 7b). The plain (cell-free) Algi/zein scaffold displayed an irregular porous network with a heterogeneous surface texture. After the C2C12 culture, additional fibrous features were observed on the scaffold surface (Figure S3a). In contrast, the plain Algi/zein ^(AP:MP)^ scaffold showed a more aligned fibrous architecture. Following C2C12 culture in Algi/zein ^(AP:MP)^, these fibrous structures were more pronounced, and aligned bundle-like features were observed (Figure 7b). Similar morphologies were detected across different fields of view (Figure S3b). Overall, these results indicate that AP/MP incorporation was associated with increased surface alignment and structural organization of the scaffold after culture.

Immunofluorescence staining of myosin demonstrated differential myogenic activity between Algi/zein and Algi/zein ^(AP:MP)^ (Figure 7c). In the Algi/zein ^(AP:MP)^ scaffold, myosin expression displayed highly aligned myotube formation with abundant nuclei, indicative of mature myotube structure. Conversely, the Algi/zein (free powder) scaffold showed weak and disorganized myosin expression, with no evidence of aligned myotube structures. The Algi/zein ^(AP:MP)^ scaffold supports robust myotube alignment without external molds, indicating that morphological organization represents the primary demonstrable outcome under the present conditions.

#### 3.6.3. Metabolite Profile of Beef, Composite Algi/Zein, and Algi/Zein ^(AP:MP)^ Scaffolds

**Figure 8:**
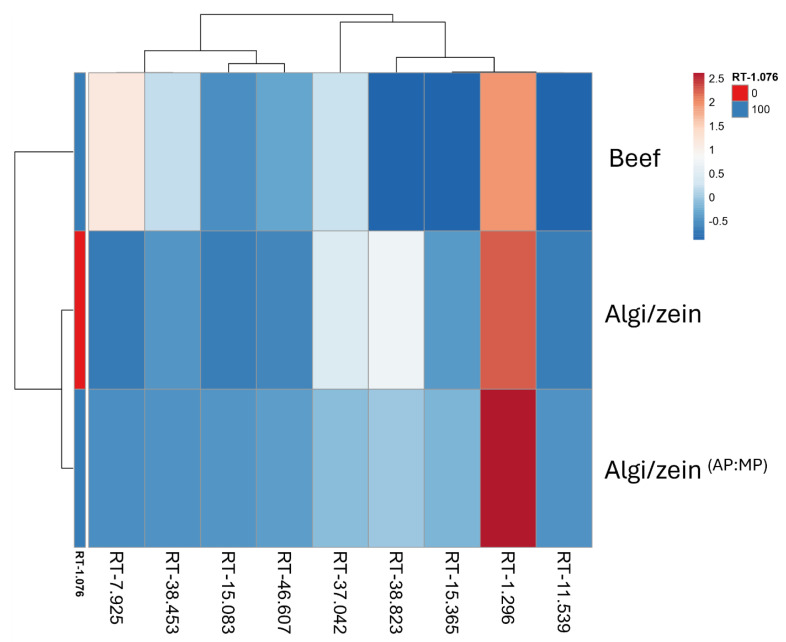
Heatmap analysis of metabolic profile in beef, Alginate-zein (Algi/zein), and Algi/zein supplemented with adipocyte (AP) and myotube powder (MP) (Algi/zein ^(AP:MP)^). Hierarchical clustering and heatmap visualization of metabolites detected at various retention times (RT) illustrate the biochemical differences among beef, Algi/zein, and Algi/zein ^(AP:MP)^ samples. Metabolite intensities were normalized and scaled (z-score) across rows. Each row represents a metabolite (identified by RT), and each column represents a sample group. Distinct clustering patterns indicate that Algi/zein ^(AP:MP)^ shares more metabolomic similarities with beef than the plain Algi/zein scaffold, particularly at RT 1.076 and RT 1.296, suggesting the successful incorporation of bioactive compounds from AP and MP. Color gradient represents relative abundance, with red indicating high and blue indicating low intensity.

A hierarchical clustering heatmap was generated to compare metabolite profiles among beef, composite Algi/zein, and Algi/zein ^(AP:MP)^ scaffolds. The retention time (RT) values displayed distinct clustering patterns, indicating putative differences in metabolite composition across the groups (Figure 8). The Algi/zein ^(AP:MP)^ scaffold exhibited stronger signals at RT-1.296 and RT-1.076, approaching those of beef, suggesting the putative presence of metabolites associated with muscle tissue. In contrast, the Algi/zein scaffold showed moderate signals at RT-1.296 and RT-3.7042, with minimal activity at RT-1.076. Beef samples clustered separately, exhibiting a broader range of RT intensities, including high signals at RT-7.925 and RT-1.296, reflecting their native metabolic complexity.

All peak assignments are putative (MSI Level 3) based on previously published retention times (Table S1), without confirmation by authentic standards. These results suggest that incorporation of AP and MP components alters the volatile metabolite profile of the scaffold toward patterns that partially overlap with beef, based on putative RT-based assignments.

## 4. Discussion

This study demonstrates that incorporating MP and AP powders into Algi/zein hydrogels can generate food-relevant scaffolds that partially mimic muscle-related structural and biochemical features in vitro. The composite scaffolds supported C2C12 alignment and myotube formation, and RT-based volatile profiling showed partial overlap with beef patterns. These findings suggest MP/AP supplementation as a potential strategy to enhance scaffold bioactivity; however, further validation under scalable cultured-meat processing conditions is required.

From a materials perspective, the integration of zein into Algi matrices provided a scaffold with reduced swelling capacity and showed biodegradability. Previous studies have reported that scaffolds with moderate degradation rates support cell proliferation and differentiation while allowing for gradual matrix turnover, which is crucial for tissue maturation in cultured-meat systems. For instance, scaffolds composed of natural biopolymers and plant components typically decay more quickly than synthetic and composite scaffolds, which can be advantageous for muscle tissue engineering [39,50]. This behavior is consistent with zein’s hydrophobic nature, which limits water uptake and slows dissolution [51,52,53]. Zein scaffolds retained internal pore connectivity, which may facilitate the diffusion of nutrients and cell infiltration [15,18,54]. Collectively, these properties support the use of Algi/zein as a scalable, food-grade platform for engineered muscle constructs.

The incorporation of cell-derived powders (MP and AP) into Algi/zein scaffolds provides lineage-specific ECM components that support myogenic differentiation and tissue organization. Adipocyte-derived ECM components have been reported to promote adipogenic differentiation and vascular network development within muscle constructs, thereby enhancing functional tissue integration [55]. In the present study, enhanced myogenic alignment of C2C12 cells in Algi/zein ^(AP:MP)^ scaffolds is likely mediated by ECM proteins such as collagen, laminin, and fibronectin, which support cell adhesion and myotube formation, together with scaffold topographical cues that guide alignment [56,57]. In addition, adipocyte-derived components may contribute paracrine signals, including bone morphogenetic proteins (BMPs), adipokines (e.g., leptin and adiponectin), and extracellular vesicles, which collectively support myotube formation, tissue organization, and functional maturation [58]. Although AP was dissolved in 0.1% DMSO for preparation purposes, this low concentration is widely tolerated in in vitro systems [59] and did not interfere with cytocompatibility or myogenic differentiation under the present experimental conditions.

In the context of cultured-meat development, several edible scaffold systems have been reported, including Algi, gelatin, soy-protein, and plant-derived porous scaffolds designed to support myoblast attachment and differentiation. Algi and gelatin-based scaffolds promote cell viability and alignment, but primarily provide physical support with limited tissue-specific biochemical signaling [27]. Soy protein and plant-based scaffolds offer food compatibility but often lack muscle or adipose-specific biological cues [60]. Compared with these reported systems, the present Algi/zein ^(AP:MP)^ scaffold integrates both food-grade structural support and lineage-specific biochemical components derived from muscle and adipose tissues, enabling enhanced cellular alignment and partial biochemical mimicry.

In addition, several contemporary cultured-meat approaches rely on layer-by-layer or compartmentalized scaffold architectures in which adipocyte and myocyte-laden matrices are physically assembled. However, these constructions often remain biochemically segregated, thereby restricting reciprocal biochemical signaling between muscle and adipose components. For example, gelatin- and soymilk-based scaffolds have been used to fabricate fat-containing cultured meat by stacking individually differentiated muscle and adipose-like layers, forming composite tissues. However, these layers remain biochemically distinct rather than fully integrated within a single matrix [38]. Edible 3D Algi–gelatin hydrogel scaffolds have also been developed to support cell growth in structured cultured-meat models. However, these systems primarily provide mechanical support without integrated lineage-specific biochemical cues [61]. In contrast, the present Algi/zein ^(AP:MP)^ composite scaffold integrates muscle and adipose-derived biochemical cues within a single edible matrix, enabling concurrent structural support and lineage-specific bioactivity without the need for live co-culture systems.

In our study, the inclusion of AP powder in Algi/zein scaffolds did not compromise C2C12 differentiation or alignment; rather, it provided biochemical cues that may support and enhance myotube organization. This approach avoids the complications observed in direct muscle–adipocyte co-cultures, where paracrine signaling from live adipocytes can suppress myogenesis and increase atrophy markers and inflammatory cytokines [62]. By using cell-derived powders, beneficial signals from adipocytes may be retained, creating a microenvironment that promotes myogenic differentiation. These findings highlight the benefit of combining muscle and adipose-derived components to support both structural organization and biochemical functionality.

FTIR analysis confirmed the presence of protein and lipid-associated peaks consistent with ECM-like structures. The fibrous morphology of the Algi/zein ^(AP:MP)^ scaffold provides alignment cues that may facilitate myotube maturation [63,64]. Immunofluorescence of myosin further supports that the structural anisotropy of Algi/zein ^(AP:MP)^ contributes to myogenic differentiation. While the specific biomolecules were not directly identified, prior studies indicate that similar cell-derived powders may contain proteins, peptides, and ECM fragments that plausibly enhance differentiation [36].

Metabolomic analysis revealed that the biochemical profile of the composite scaffold shared features with native beef tissue. Beef was chosen as a reference because it represents a high-protein, nutrient-rich food with a well-characterized metabolic profile, facilitating meaningful comparisons for cultured-meat applications [36]. In our study, since the experiments were conducted using a murine myoblast cell line and cell-derived powders, comparison with mouse muscle tissue may be more appropriate. We produced cell-derived powders and, as a preliminary study, optimized the DNase treatment time to effectively remove residual DNA [65]. Although the present study establishes proof-of-concept, inclusion of additional material- and species-specific control groups and extended functional validation would provide further mechanistic insight; however, such controls were technically constrained by the physicochemical requirements for stable Algi/zein hydrogel formation and will be addressed in future studies.

Recent studies have highlighted that edible scaffolds for cultured meat should not only support cellular functions but also meet requirements for scalable manufacturing, food safety compliance, and tailored rheological properties [66]. Hybrid gel-based scaffold systems, composed of naturally derived polymers such as alginate, plant proteins, and polysaccharides with food-grade functional additives, have been proposed as a promising platform that balances biocompatibility, edibility, mechanical integrity, and consumer safety, facilitating scalable production approaches such as extrusion or printing [66,67]. These hybrid materials enhance mechanical stability and rheological behavior, which are critical for maintaining structural integrity during culture and downstream processing, and align with regulatory frameworks for edible components (e.g., GRAS status) [68].

Despite these advances, several limitations should be acknowledged. This study was conducted using the murine C2C12 myoblast cell line, which may not fully recapitulate the behavior of livestock-derived muscle cells relevant to commercial cultured-meat production. Importantly, functional muscle performance, including contractility, calcium transients, or responses to electrical stimulation, was not evaluated; therefore, no conclusions regarding functional maturation were drawn. Muscle differentiation was inferred solely from morphological alignment and myosin immunostaining. Although trends were observed, the lack of qPCR analysis under the present experimental conditions indicates the need for larger sample sizes and additional markers (e.g., TnT, MHC isoforms) in future studies. In addition, food-relevant properties such as texture, rheology, diffusion characteristics, media perfusion, sensory attributes, and long-term stability were not assessed. While metabolomic profiling suggested partial biochemical similarity to native beef tissue, compound identification remained putative, limiting chemical interpretation. These limitations highlight the need for further validation using livestock-derived cells, functional assays, and comprehensive food-grade evaluations. Finally, the absence of mechanical stimulation, long-term maturation analysis, and in vivo validation, together with the use of murine rather than primary bovine myoblasts, may limit the direct translational scalability of this platform to industrial cultured-meat production.

## 5. Conclusions

This study demonstrates that MP/AP-enriched Algi/zein hydrogels enable robust intrinsic myotube alignment without external molds while maintaining cytocompatible growth of C2C12 myoblasts, representing the primary functional outcome of the scaffold system. Metabolomic profiling revealed partial chemical similarity between MP/AP-enriched Algi/zein scaffolds and native beef tissue. Hierarchical clustering was primarily driven by RT-based features, showing partial overlap with beef, while other features reflected distinct scaffold-related or processing-associated profiles, including oxidative differences. All metabolite assignments were putative (MSI Level 3), based on previously reported retention times without confirmation by authentic standards, and should therefore be interpreted with appropriate caution. Overall, these findings support the potential of MP/AP-enriched Algi/zein hydrogels as a food-grade scaffold platform for structured cultured-meat applications, rather than demonstrating chemical equivalence to native beef. Further studies employing MSI-compliant metabolomic validation and advanced quality control will be required to strengthen translational relevance.

## Figures and Tables

**Table 1:** FTIR spectral assignments and functional clues of MP, AP components, and Algi/zein, Algi/zein^(AP:MP)^ scaffolds.

Wavenumber (cm^−1^)	Functional Group	FTIR Clue	Sample(s)	Interpretation	References
~3300	O–H or N–H stretching (broad)	Protein backbone or water content	MP, AP, Algi/zein ^(AP:MP)^	Indicates the presence of proteins or moisture in biological powders and composites.	[44]
~2950–2850	C–H stretching (asymmetric/symmetric)	Lipid aliphatic chains	AP, Algi/zein ^(AP:MP)^	Prominent in AP, reflects fatty acid content.	[45]
~1650	Amide I (C=O stretch of peptide bond)	Protein secondary structure (α-helix/β-sheet)	MP, Algi/zein, and Algi/zein ^(AP:MP)^	Confirms protein presence from the MP component and partial retention in the scaffold. No secondary structure analysis performed.	[44]
~1540	Amide II (N–H bending + C–N stretching)	Protein secondary structure	MP, Algi/zein ^(AP:MP)^	Strong in MP, slightly retained in the scaffold with biological additives.	[44]
~1450–1400	CH_2_ bending	Lipid or carbohydrate content	AP, Algi/zein ^(AP:MP)^	Reflects the contribution of lipids from adipocytes.	[46]
~1240–1020	C–O stretching (polysaccharide region)	Alginate or carbohydrate fingerprint	Algi/zein, and Algi/zein ^(AP:MP)^	The signature of Algi is more intense with scaffold materials.	[47]
~1730	C=O stretch (ester/carboxyl group)	Lipid esters or alginate carboxylate	Algi/zein, and Algi/zein ^(AP:MP)^	Indicates fatty esters or Algi crosslinks.	[45]
~875–800	C–H deformation/fingerprint region	Structural scaffold features or polysaccharide markers	Algi/zein, and Algi/zein ^(AP:MP)^	Unique to zein and scaffold interactions.	[48,49]

## Data Availability

The datasets generated and analyzed during the current study are included in the article and its Supplementary Materials. Additional data are available from the corresponding author upon reasonable request.

## Supplementary Materials

The following supporting information can be downloaded at: https://www.mdpi.com/article/10.3390/foods15030522/s1, Figure S1: C2C12 cell viability on 2D culture through growth media supplemented with different concentrations of myotube powder (MP) on days 1, 3, and 5. (a) Live/dead staining of C2C12 cells through growth media containing MP with different concentrations (0.2,0.3, and 0.4 μg/µL). Live cells are stained green, and dead cells are stained red. Scale bars 100 μm. (b) Cell viability % using the CCK-8 assay was analyzed at the same time as the cell culture. All data are presented as mean ± SE. (n = 8). Data was analyzed using a two-way ANOVA with MP concentration and time as factors, followed by Šidák-corrected post-hoc comparisons between concentrations at each time point. Significance was denoted **** *p* < 0.00012; Figure S2. Additional fields of desmin expression in 2D C2C12 cells cultured in differentiation media supplemented with myotube powder (MP). C2C12 cells were differentiated through media containing 0.01, 0.05, and 0.1 µg/µL MP for 7 days. Cells were fixed and stained for desmin (green), a muscle-specific intermediate filament marker, and counterstained with DAPI (blue) for nuclei. Increased MP concentration promoted alignment and elongation of myotubes, especially at 0.05 µg/µL and 0.1 µg/µL, while structural integrity was altered at 0.1 µg/µL, indicating possible scaffold remodeling or contraction. Scale bar 100 µm. Figure S3. Additional fields of SEM analysis of Alginate-zein (Algi/zein) scaffolds supplemented with Adipocyte (AP) and myotube powder (MP) (Algi/zein ^AP:MP^) after 10 days of cell differentiation. (a) Algi/zein shows few ECM (red dashed circles), indicating signs of cellular differentiation. (b) More ECM in Algi/zein ^(AP:MP)^ hydrogel indicates the porous network with interconnected fibers, supporting cell infiltration. Cigar-like morphology of aligned myotubes was distinguished at Algi/zein ^(AP:MP)^ hydrogel, facilitating organized myotube formation. Scale bars 10 μm. Table S1. List of volatile compounds detected by GC–MS in scaffold and beef samples [69,70,71].

## Abbreviations

Algi	Alginate
Algi/zein	Alginate/zein
AP	Adipocyte powder
MP	Myotube powder
Algi/zein ^AP:MP^	Alginate-zein hydrogel coated with AP and MP components
RT	Retention Time
