# Supplementary material for: Myotube/Adipocyte Powder-Enriched Alginate–Zein Hydrogels Support Myotube Alignment for 3D Myoblast Culture
Source: Foods. 2026 Feb 2;15(3):522. doi: 10.3390/foods15030522 (PMC12897132; doi:10.3390/foods15030522)
Supplement: Supplementary file 1 [file foods-15-00522-s001.zip › foods-4049716-supplementary.docx]

**
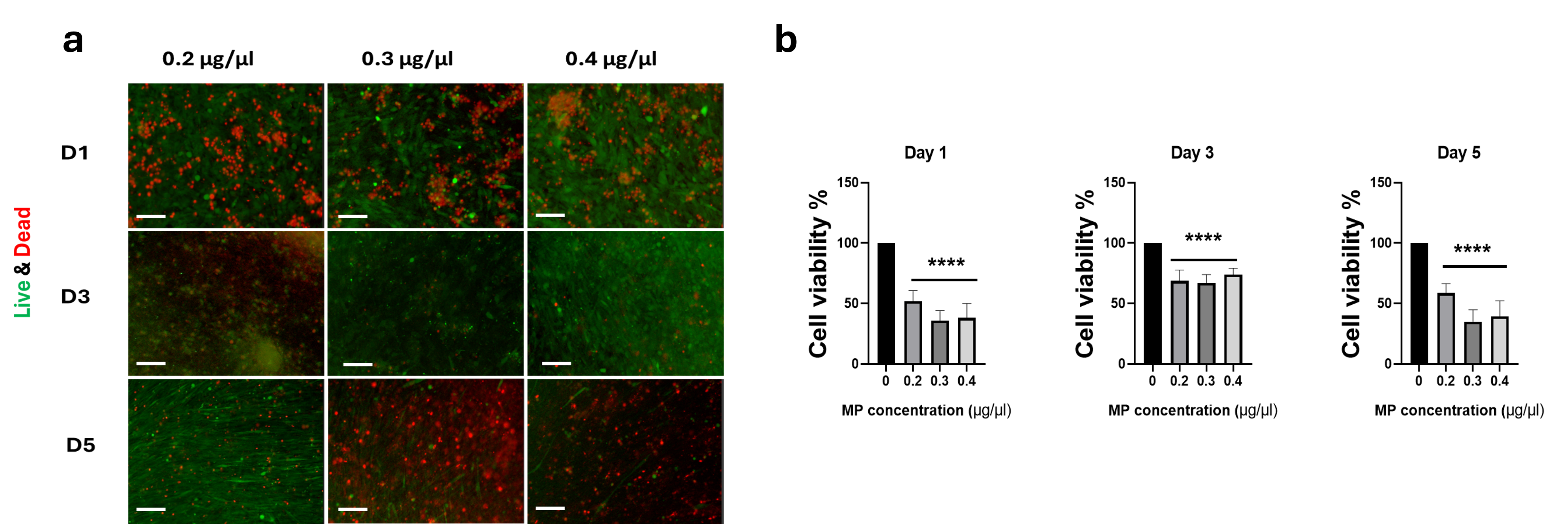
**

**Figure S1.** C2C12 cell viability on 2D culture through growth media supplemented with different concentrations of myotube powder (MP) on days 1, 3, and 5. (a) Live/dead staining of C2C12 cells through growth media containing MP with different concentrations (0.2,0.3, and 0.4 μg/µL). Live cells are stained green, and dead cells are stained red. Scale bars 100 μm. (b) Cell viability % using the CCK-8 assay was analyzed at the same time as the cell culture. All data are presented as mean ± SE. (n = 8). Data was analyzed using a two-way ANOVA with MP concentration and time as factors, followed by Šidák-corrected post-hoc comparisons between concentrations at each time point. Significance was denoted ****P < 0.00012.

**
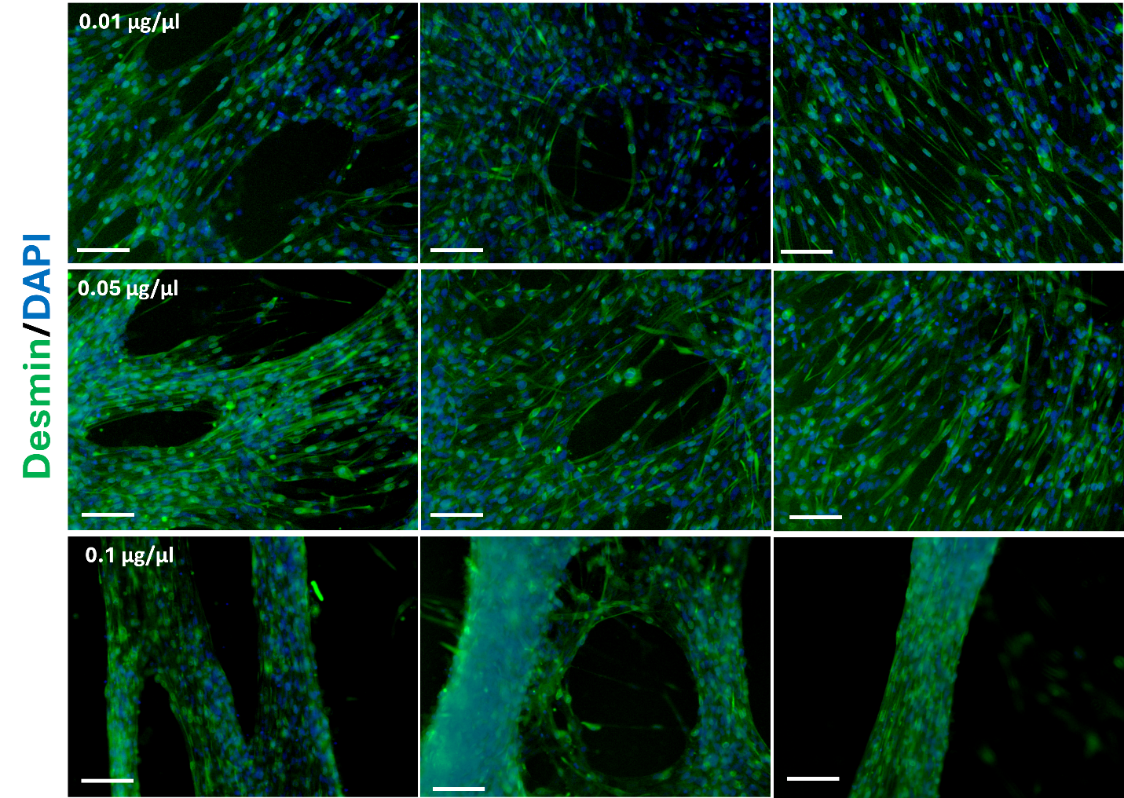
**

**Figure S2.** Additional fields of desmin expression in 2D C2C12 cells cultured in differentiation media supplemented with myotube powder (MP). C2C12 cells were differentiated through media containing 0.01, 0.05, and 0.1 µg/µL MP for 7 days. Cells were fixed and stained for desmin (green), a muscle-specific intermediate filament marker, and counterstained with DAPI (blue) for nuclei. Increased MP concentration promoted alignment and elongation of myotubes, especially at 0.05 µg/µL and 0.1 µg/µL, while structural integrity was altered at 0.1 µg/µL, indicating possible scaffold remodeling or contraction. Scale bar 100 µm.

**
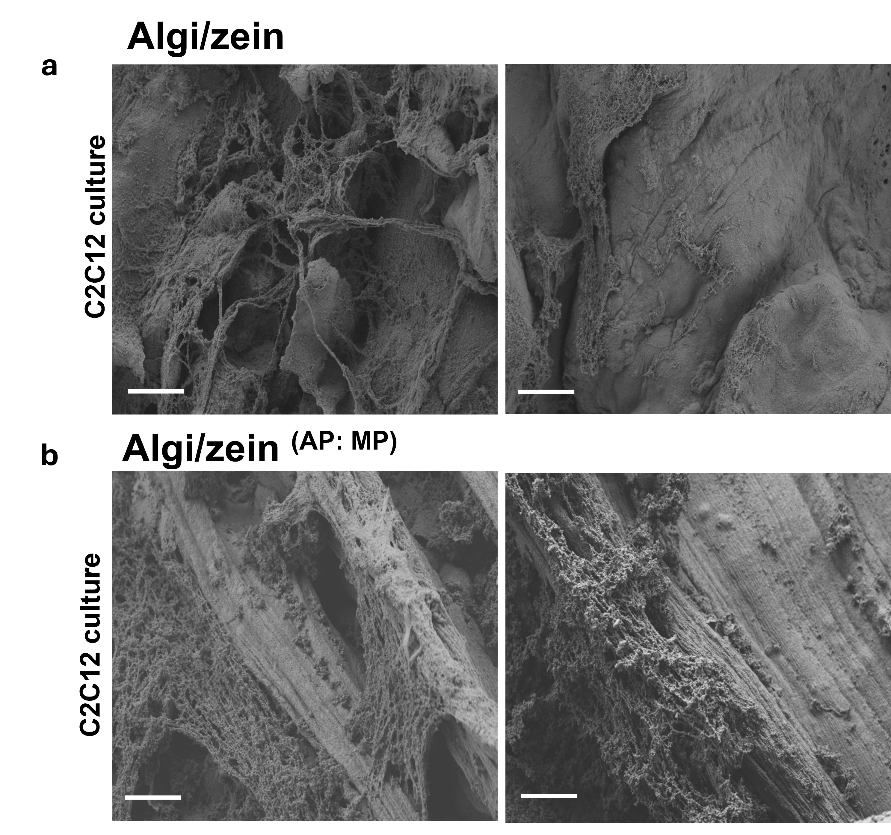
**

**Figure S3**. Additional fields of SEM analysis of Alginate-zein (Algi/zein) scaffolds supplemented with Adipocyte (AP) and myotube powder (MP) (Algi/zein ^AP: MP^) after 10 days of cell differentiation. (a) Algi/zein shows few ECM (red dashed circles), indicating signs of cellular differentiation. (b) More ECM in Algi/zein^(AP: MP)^ hydrogel indicates the porous network with interconnected fibers, supporting cell infiltration. Cigar-like morphology of aligned myotubes was distinguished at Algi/zein^(AP: MP)^  hydrogel, facilitating organized myotube formation. Scale bars 10 μm.

**Table S1**. List of volatile compounds detected by GC–MS in scaffold and beef samples.

| Rt(minutes) | Putative compounds | Algi/zein | Algi/zein ^(AP:MP)^ | Beef | Interpretation | References |
| --- | --- | --- | --- | --- | --- | --- |
| RT-1.076 | Acetaldehyde | 0 | 100 | 100 | Found in fresh meat, due to lipid oxidation or fermentation, suggesting an active aldehyde pathway | (1) |
| RT-1.296 | Ethanol | 100 | 100 | 40.66 | Detected in all samples. Ethanol was used only as a transient solvent during zein dissolution  and was removed prior to cell culture and GC–MS analysis. Therefore, ethanol signals should  not be interpreted as intrinsic scaffold-derived metabolites and likely reflect background or  sample-processing-related traces. | (1) |
| RT-7.925 | Hexanal | 1.52 | 1.01 | 29.29 | Key aldehyde from Linoleic acid oxidation, a major freshness indicator in meat | (2) |
| RT-11.539 | Nonanal | 1.69 | 1.5 | 0 | Longer chain aldehyde, minor in Algi/zein samples and absent in beef, may be due to breakdown | (3) |
| RT-15.083 | 2,3-Octanedione | 2.54 | 3.21 | 5.15 | Ketone, common in dairy and meat, suggests Maillard or lipid degradation | (1) |
| RT-15.365 | Benzaldehyde | 10.76 | 11.96 | 0 | Almond-like aroma derived from plant phenolics, strong in Algi/zein samples | (3) |
| RT-37.042 | Octanal | 40.25 | 13.78 | 16.1 | Citrus-smelling aldehyde, derived from Oleic acid oxidation, is high in Algi/zein samples and also present in beef | (2) |
| RT-38.453 | 1-Octen-3ol | 9.21 | 2.14 | 15.56 | Mushroom-like aroma, a marker of lipid oxidation in meat, is high in beef | (3) |
| RT-38.823 | 2-Nonanone | 49.43 | 17.72 | 0 | Ketone from fatty acid degeneration, abundant in Algi/zein samples | (1) |
| RT-46.607 | 2-Pentylfuran | 4.59 | 4.93 | 8.29 | Lipid oxidation contributes to green/beany notes. More prominent in beef. | (3) |

All metabolite identifications are putative (MSI Level 3) and were assigned based on previously reported retention times. No authentic standards, MS/MS fragmentation, or library match scores were obtained

1. Wang X, Zhu L, Han Y, Xu L, Jin J, Cai Y, et al. Analysis of volatile compounds between raw and cooked beef by HS‐SPME–GC–MS. Journal of Food Processing and Preservation. 2018;42(2):e13503.

2. Vilar EG, O'Sullivan MG, Kerry JP, Kilcawley KN. Volatile organic compounds in beef and pork by gas chromatography‐mass spectrometry: A review. Separation Science Plus. 2022;5(9):482-512.

3. Zhan P, Tian H, Sun B, Zhang Y, Chen H. Quality control of mutton by using volatile compound fingerprinting techniques and chemometric methods. Journal of Food Quality. 2017;2017(1):9273929.
